# Intra- and inter-host evolution of H9N2 influenza A virus in Japanese quail

**DOI:** 10.1093/ve/veac001

**Published:** 2022-01-08

**Authors:** Lucas M Ferreri, Ginger Geiger, Brittany Seibert, Adebimpe Obadan, Daniela Rajao, Anice C Lowen, Daniel R Perez

**Affiliations:** Department of Population Health, Poultry Diagnostic and Research Center, College of Veterinary Medicine, University of Georgia, 953 College Station Rd, Athens, GA 30602, USA; Department of Microbiology and Immunology, Emory University School of Medicine, Atlanta, GA 30322, USA; Department of Population Health, Poultry Diagnostic and Research Center, College of Veterinary Medicine, University of Georgia, 953 College Station Rd, Athens, GA 30602, USA; Department of Population Health, Poultry Diagnostic and Research Center, College of Veterinary Medicine, University of Georgia, 953 College Station Rd, Athens, GA 30602, USA; Amazon.com, Seattle, 1510 Clifton Rd, WA, USA; Department of Population Health, Poultry Diagnostic and Research Center, College of Veterinary Medicine, University of Georgia, 953 College Station Rd, Athens, GA 30602, USA; Department of Microbiology and Immunology, Emory University School of Medicine, Atlanta, GA 30322, USA; Department of Population Health, Poultry Diagnostic and Research Center, College of Veterinary Medicine, University of Georgia, 953 College Station Rd, Athens, GA 30602, USA

**Keywords:** influenza, positive selection, transmission bottleneck, clonalinterference, poultry, virus population

## Abstract

Influenza A viruses (IAVs) are constantly evolving. Crucial steps in the infection cycle, such as sialic acid (SA) receptor binding on the host cell surface, can either promote or hamper the emergence of new variants. We previously assessed the relative fitness in Japanese quail of H9N2 variant viruses differing at a single amino acid position, residue 216 in the hemagglutinin (HA) viral surface protein. This site is known to modulate SA recognition. Our prior study generated a valuable set of longitudinal samples from quail transmission groups where the inoculum comprised different mixed populations of HA 216 variant viruses. Here, we leveraged these samples to examine the evolutionary dynamics of viral populations within and between inoculated and naïve contact quails. We found that positive selection dominated HA gene evolution, but fixation of the fittest variant depended on the competition mixture. Analysis of the whole genome revealed further evidence of positive selection acting both within and between hosts. Positive selection drove fixation of variants in non-HA segments within inoculated and contact quails. Importantly, transmission bottlenecks were modulated by the molecular signature at HA 216, revealing viral receptor usage as a determinant of transmitted diversity. Overall, we show that selection strongly shaped the evolutionary dynamics within and between quails. These findings support the notion that selective processes act effectively on IAV populations in poultry hosts, facilitating rapid viral evolution in this ecological niche.

## 1. Introduction

Understanding the evolutionary processes shaping virus populations is essential in increasing our knowledge on the factors mediating host switching, immune evasion, and drug resistance, among other phenotypic changes. The evolution of RNA viruses is linked to their intrinsic capacity to generate large and diverse populations within the infected host (Parvin et al. 1986; Crotty et al. 2000; Vignuzzi et al. 2006; Aaskov et al. 2006). The advent of next generation sequencing allows for systematic and detailed characterization of virus populations and their evolution. Studies of intra- and inter-host virus evolution are invaluable for determining the extent to which global processes are recapitulated at within- and between-host scales (Lauring 2020). Once infection of the host occurs, the error-prone replication of RNA viruses produces populations that are subject to selective and stochastic forces that dictate the viruses’ evolutionary fate (Moya, Holmes, and Gonzalez-Candelas 2004). Positive selection is the deterministic process in which new genetic variants sweep a population by offering a fitness advantage (Rouzine, Rodrigo, and Coffin 2001). Alternatively, negative or purifying selection can act on variants that negatively impact fitness and are purged from the population. On the other hand, genetic drift is the stochastic process in which new variants can reach high frequencies by chance. Even though positive selection is the main evolutionary force acting on seasonal influenza viruses at global scale (Fitch et al. 1991, 1997; Bedford et al. 2015), studies of intra-host infection have suggested that positive selection does not strongly influence evolution at this scale (Debbink et al. 2017; McCrone et al. 2018; Han, Maurer-Stroh, and Russell 2019). Influenza viruses affecting poultry species go through similar global evolutionary processes to those circulating in humans (Lee et al. 2016), but less is known about viral dynamics at the intra- and inter-host level.

H9N2 viruses are enzootic in most of Asia, the Middle East, and parts of Africa, where they have caused disease outbreaks in chickens, quail, and other minor poultry species (Wan and Perez 2007). H9N2 viruses contributed gene segments to the goose/Guangdong H5N1 lineage and the Asian-lineage H7N9 and H10N8 viruses, all of which have caused human fatalities (Guan et al. 1999; Lam et al. 2013; Tharakaraman et al. 2013; Chen et al. 2014; Pu et al. 2015). Human infections with H9N2 subtype influenza A virus (IAV) have been reported, showing that these viruses can cross the avian–mammalian host barrier and therefore present a pandemic concern (Peiris et al. 1999; Butt et al. 2005; Cheng et al. 2011; Pan et al. 2018).

A critical component of the avian–mammalian host barrier is the limited availability of sialic acid (SA) receptors used by avian IAV in mammalian hosts. Changes to the viral receptor binding site in HA are therefore often needed to mediate host switching. We have shown that amino acid 216 in the hemagglutinin (HA) of the H9 subtype (226 in H3 subtype HA numbering) is a major determinant in the recognition of terminal SA on host cell surfaces (Wan and Perez 2006, 2007). H9 strains that carry HA L216 preferentially bind terminal SAs in an α2-6 conformation (α2-6SA), typically found in the upper respiratory tract of humans and of some gallinaceous bird species (Wan and Perez 2006; Kimble, Nieto, and Perez 2010). In contrast, H9 strains that carry HA Q216 bind α2-3SA, more typically found in the intestinal tracts of waterfowl and land-based poultry (Wan and Perez 2007). Looking across all available sequences, the majority of H9 field isolates (typically associated with the N2 NA subtype) contain either L216 (>80 per cent) or Q216 (∼18 per cent) and a small proportion (<2 per cent) contain M216, I216, F216, or S216. Of note, however, a temporal trend is apparent, with older avian H9N2 virus isolates having the HA Q216 residue and the vast majority of recent H9N2 strains carrying HA L216 (Matrosovich, Krauss, and Webster 2001; Choi et al. 2004).

Using the prototypic strain A/guinea fowl/Hong Kong/WF10/1999 (H9N2) (WF10), we previously explored the plasticity of amino acid position 216 in the H9 HA (Obadan et al. 2019). In this prior work, we conducted a replication–transmission study in Japanese quail, a natural host of H9N2 IAVs that circulate in poultry. Four groups of inoculated quail were placed in direct contact with naïve quail to test the relative fitness and transmission of virus variants differing only at amino acid 216 in the HA. These studies revealed significant tolerance of position 216 to accept multiple different amino acids. In Japanese quail, L216 and, to a lesser extent, Q216 were favored for replication and transmission.

Taking advantage of the longitudinal nature of this previous study, herein we performed a comprehensive analysis of the dynamics of variant frequencies and genome-wide evolution during replication and transmission. We show that selective processes act in concert, shaping the virus population within and between quails. We also observed that specific variants elsewhere in the genome have similar dynamics across individuals, independently of the molecular marker at HA 216, strongly implicating common selective pressure, efficient reassortment, and lack of genetic linkage. Furthermore, we found that transmission bottlenecks were modulated by the molecular signature at HA 216, revealing a role for viral receptor specificity in shaping the transfer of viral genetic diversity between hosts.

## 2. Materials and methods

### 2.1 Ethics statement and Quail housing

All animal studies adhered to the Institutional Animal Care and Use Committee (IACUC) guidebook of the Office of Laboratory Animal Welfare (59) and PHS policy on the humane care and of use of laboratory animals. Studies were conducted under animal biosafety level 2 (ABSL-2) containment and approved by the IACUC of the University of Georgia (protocol A201506-026-Y3-A5). Animals were humanely euthanized following guidelines approved by the American Veterinary Medical Association. Quails were maintained in isolators with dimensions of 42" length, 24" width and 31" height. Water and food were provided ad libitum.

### 2.2 Generation of 216 H9N2 virus library

In our previous report (Obadan et al. 2019), virus rescue experiments were performed using PCR reverse genetics (PCR-RG), as previously described (Chen et al. 2012; Perez et al. 2020). To generate the HA 216 variants, the corresponding mutant PCR-RG amplicons were co-transfected along with seven reverse genetics plasmids encoding the rest of the WF10 genome. For transfection, co-cultures of MDCK and 293T cells were seeded in each well of a six-well plate overnight at 37ºC. The following day, 1 µg of each of the seven plasmids of WF10 and 1 µg of PCR-RG amplicon was mixed with 16 µl of TransIT-LT1 transfection reagent (Mirus Bio LLC, Madison, WI) and incubated for 45 min. After 45 min, the MDCK/293T cells were overlaid with transfection mixture and incubated at 37ºC for 24 h. At 24 h post-transfection, the transfection mixture was replaced with fresh Opti-MEM I media (Life Technologies, Carlsbad, CA) containing 1 µg/ml of tosylsulfonyl phenylalanyl chloromethyl ketone (TPCK) treated-trypsin (Worthington Biochemicals, Lakewood, NJ) and 1 per cent ATB/ATM (Sigma-Aldrich, St. Louis, MO). Supernatants containing rescued viruses were collected at 96 h post-transfection.

### 2.3 Isolation and identification of individual virus variants and growth of virus stocks

Virus variants (var) in the virus libraries were isolated by limiting dilution assays as previously described (Kimble et al. 2014; Obadan et al. 2019). MDCK cells (2×10^4 cells/well in ninety-six well plate) were infected with eight serial 10-fold dilution of the rescued virus library in Opti-MEM I media (Life Technologies, Carlsbad, CA) containing 1 µg/ml of TPCK treated-trypsin and 1 per cent ATB/ATM. The var strains were produced starting from the variant virus library. After 72 h incubation at 37ºC, var virus supernatants were collected from wells infected with the most diluted sample displaying the cytopathic effect. This process was repeated once, followed by Sanger sequencing to determine the amino acid at position 216 in HA. A third round of limiting dilution was carried out for samples yet to resolve at position 216 after the second limiting dilution. The var viruses were further expanded in MDCK cells and stocks aliquoted and stored at −80ºC until used. The var virus stocks were titrated by 50 per cent tissue culture infectious dose (TCID50) in MDCK cells and titers determined by the Reed–Muench method (Reed and Muench 1938).

### 2.4 In vitro RNA production for error analysis of variant detection

To evaluate intrinsic error of the variant detection workflow we generated a non-influenza RNA template. The beta lactamase gene (β-lac) was selected because it has ∼50 per cent GC content. In vitro β-lac RNA transcription was performed with the T7 RiboMAX^TM^ Express Large Scale RNA Production System (Promega, Fitchburg WI) according to manufacturer instructions. RNA was measure using Qubit^TM^ RNA HS Assay Kit (Thermo Fisher Scientific, Waltham, MA). Primers to amplify the β-lac gene were designed to contain UTR sequences recognized by Opti primers used for influenza virus gene amplification: Opti-BlacFor 5ʹ AGCRAAAGCAGGATGAGTATTCAACATTTCCGTGTCG 3ʹ, Blac-Opti-T7-clampRev 5ʹ CAGAGATGCATAATACGACTCACTATAGGAGTAGAACCAAGGTTACCAATGCTTAATCAGTGAGGCACC 3ʹ. Amplification was performed following the IAV whole-genome amplification multi-segment RT-PCR (MS-RTPCR) as previously described (Mena et al. 2016). The β-lac gene was sequenced using the next generation sequencing approach described below.

### 2.5 Defining cutoff for variant analysis

For these analyses, we defined the intrinsic error of the workflow by using an artificial 861-nt in vitro-transcribed RNA derived from the β-lactamase (β-lac) gene with a GC content of 49.1 per cent. Using an artificial RNA allowed us to circumvent the virus RNA-dependent RNA polymerase’s error, giving a direct and clean readout of the NGS workflow error. RT-PCR amplification of the β-lac RNA was performed using specific primers flanked by the UTR sequences derived from the IAV genome to more faithfully mimic conditions used for the MS-RTPCR amplification and the NGS conditions for IAV whole genome sequencing (Mena et al. 2016) (Supplementary Fig. S1). NGS of three independent amplifications revealed that artifactual β-lac variants had a mean frequency of 0.0016 ± 0.0007. Based on this observation, variant sites were called as such if they were present with at least 400X coverage and a frequency ≥0.02 equivalent to ∼12.5X the mean background noise.

### 2.6 MS-RTPCR amplification and next generation sequencing

Whole-genome sequencing of swab samples was performed as previously described (Ferreri et al. 2019). Briefly, RNA was extracted using the RNeasy mini kit (Qiagen, Valencia, CA) or a MagNA Pure LC RNA isolation kit (Roche Life Science, Mannheim, Germany). Virus RNA was amplified in a one-step reverse transcriptase PCR for MS-RTPCR (Mena et al. 2016). Amplicon sequence libraries were prepared using the Nextera XT DNA library preparation kit (Illumina, San Diego, CA) according to the manufacturer’s protocol. Barcoded libraries were multiplexed and sequenced on a high-throughput Illumina MiSeq sequencing platform (Illumina) in a paired-end 150-nucleotide run format using MiSeq Reagent Micro Kit v2 (300 cycle) (Illumina). De novo genome assembly was performed as described previously (Mena et al. 2016).

### 2.7 Targeted NGS sequencing

We prepared Illumina libraries for targeted sequencing of a 229 base region flanking position 226 in the HA. After RNA extraction, amplicons were prepared by one-step RT-PCR amplification of viral RNA using Platinum SuperScript III One-Step RT-PCR System with Platinum Taq High Fidelity DNA Polymerase (ThermoFisher, Waltham, MA). To facilitate subsequent indexing, conserved adaptor sequences flanking the H9 HA targeted region were incorporated into the H9 HA primer set (H9 HA 849R AGACGTGTGCTCTTCCGATCTGGCTCCCTCCTGAAAGAACG and H9 HA 620F ACACTCTTTCCCTACACGACGCTCTTCCGATCTTACCGAACAAAC-AAATTTGTACA. In bold are shown the sequence corresponding to the adapter). Following amplification of the targeted region, the PCR products were cleaned using AMPure XP beads (Beckman Coulter, Indianapolis, IN) at a 0.7X ratio to eliminate fragments <250 bp and fragment size was checked on the Agilent Bioanalyzer (Santa Clara, CA). Amplicons were normalized to 0.5 ng/μL and indexes and terminal complementary flow cell oligos were added in a second PCR using NEBNext® High-Fidelity 2X PCR Master Mix (Ipswich, MA). The PCR was carried using a combination of NGS F and a single end indexing primer (Supplementary Table S1). Cycling conditions for the indexing PCR were set to 98°C for 30 s, eight cycles of 98°C for 8 s, 65°C for 30 s, and 72°C for 20 s, and then a final extension for 2 min at 72°C. The resulting libraries were cleaned using AMPure XP beads (Beckman Coulter, Indianapolis, IN) at a 0.7X ratio to eliminate fragments <250 base pairs. The final libraries were pooled and diluted following Illumina’s loading protocol.

### 2.8 In vivo competition study

The animal study has been previously described (Obadan et al. 2019). Japanese quail (4–5 weeks old) sero negative for IAV were assigned to one of five different groups (*n* = 6/group) and subsequently inoculated with HA 216 variant mixture of H9N2 viruses on the A/guinea fowl/Hong Kong/WF10/1999 (H9N2) background (10^6^ TCID_50_, 1 ml of inoculum, 0.25 ml administered via the trachea, 0.25 ml via the nares, and 0.5 ml via the cloaca). Each variant in the inoculum was calculated at the same TCID50 dose. Quail in group 1 served as a negative control and were inoculated with 1 ml of PBS. Group 2 (varΔLQ) quail were inoculated with a virus mixture containing the following 10 variant viruses: I216, S216, T216, M216, H216, N216, F216, V216, C216, and G216 viruses. Group 3 (var + Q) quails received a mixture of eleven variant viruses (the 10 variant viruses of group 2 plus the Q216 variant virus). Group 4 (var + L) is similar to Group 3, except that in the virus mixture the Q216 variant was replaced with the L216 (wt WF10) variant. Group 5 (var + LQ) was inoculated with the 12 HA 216 variants available. At day 2 post-infection (dpi), naïve quails (*n* = 6/group) were introduced as direct contacts to determine variant virus transmission. Tracheal and cloacal swabs were collected daily from each bird until 14 dpi. Swabs were suspended in 1 ml of 3.7 per cent Brain Heart infusion media (BHI) (Becton Dickinson, Sparks, MD) containing 10,000U penicillin, 10 mg of streptomycin, and 25 g/mL amphotericin B and stored at −80ºC until used in virus titrations. At 5 dpi, three quails from each group of directly inoculated quail were randomly selected and sacrificed for evaluation of virus titers in tissue samples. Likewise, at 5 days post-contact (dpc), three quails from each group of contact quail were sacrificed to establish virus titers in tissue samples.

### 2.9 Variant analysis

Analysis of non-consensus variants was made using LoFreq (Wilm et al. 2012) following the Genome Analysis Toolkit best practices (Van der Auwera et al. 2013). After removing adapters using Cutadapt (version 2.8), reads were mapped back to their reference sequence using the option mem from BWA (Li and Durbin 2009). Data formatting for GATK was made using Picard (http://broadinstitute.github.io/picard/). The use of MarkDuplicates from Picard was avoided as per LoFreq FAQs suggestion (https://csb5.github.io/lofreq/faq/) since samples were PCR products. Reads were realigned using RealignerTargetCreator and IndelRealigner from GATK. The quality of bases was recalculated using BaseRecalibrator from GATK. The resulting bam file was used to perform variant calling analysis by LoFreq. Only variants at a frequency of 0.02 with a coverage equal or above 400 were used. For detection of synonymous and nonsynonymous mutations we used the program SNPdat (Doran and Creevey 2013). Nucleotide position 4 in all segments was ignored since primers for MS-RTPCR are degenerate at this position and therefore not suitable for variant calling analyses.

### 2.10 Diversity calculation

The π statistic for measuring nucleotide diversity was calculated using the synonymous (πS) and nonsynonymous (πN) nucleotide diversity using SNPGenie (Nelson, Moncla, and Hughes 2015), which adapts Nei and Gojobori’s (1986) method of estimating synonymous and nonsynonymous substitutions for next-generation sequencing data (Nei and Gojobori 1986; Wilker et al. 2013). The cutoff utilized for π calculation was set at 0.02 of frequency. Shannon entropy was used to analyze the more complex ensemble of variants at HA 216. For this analysis, the calculation of Shannon entropy was performed after filtering out variants represented by less than 10 reads. Shannon entropy was calculated using the vegan package version 2.5 in R (Oksanen 2013).

### 2.11 Plots

All figures were made using the RStudio and the package ggplot2 (Wickham 2016) and aesthetically modified using Inkscape v0.48.1 (https://inkscape.org).

## 3. Results

### 3.1 In vivo quail experiment

We previously determined the relative fitness of a set of WF10 (H9N2) variants that differ at HA amino acid position 216 in Japanese quail (*Coturnix japonica*)(Obadan et al. 2019). Groups of quail (*n* = 6/group) were inoculated (i-qa) with one of the following virus mixtures, with each variant included at the same infectious dose. The varΔLQ inoculum contained ten variant viruses: I216, S216, T216, M216, H216, N216, F216, V216, C216, and G216. The var + Q inoculum contained the variants in the varΔLQ plus the Q216 variant. The var + L inoculum contained the variants in the varΔLQ plus the L216 variant. The var + LQ inoculum contained the varΔLQ variants plus the L216 and Q216 variants. On day 2 post-inoculation (dpi), naïve quails (*n* = 6/group) were introduced in direct contact (c-qa) with the inoculated quail to monitor virus transmission. Tracheal swabs were collected from all birds every other day (Supplementary Fig. S2). Whole influenza virus genome sequencing was performed using vRNAs extracted directly from the swab samples.

### 3.2 Intra-host dynamics of HA 216 variants are dominated by positive selection but L216 fixation was dependent on the competition mixture

**Figure 1.**
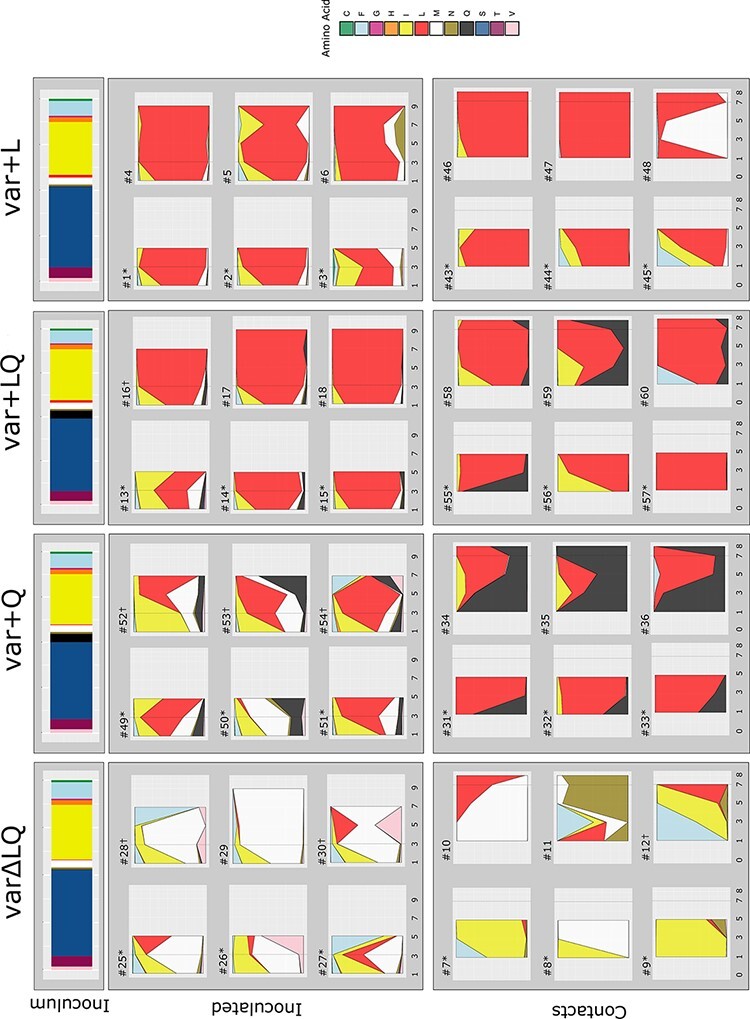
Time series analysis of position HA 216 show that L216 is rapidly selected, but the dynamics depends on the competitors present in the inoculum mixture. In the top panel, the bars show the proportions of HA216 variants present in the inoculum. Below, the stacked area plots represent frequency of amino acids present in tracheal swab samples of inoculated and contact quails. Groups are designated as varΔLQ for mix of variant viruses in absence of L and Q at 216, var + L mix of variants viruses including L216, var + Q mix of viruses including Q216 and var + LQ mix of viruses including L216 and Q216. The specimens collected at 1, 3, 5, and 7 dpi from inoculated quails and 1, 3, 5, 7, and 8 dpc (*x*-axis) from contact quails were sequenced by NGS, and the amino acid frequency (*y*-axis) was calculated. Each plot represents a single quail (Q#) and each color represents a variant at position HA 216. Dotted grey lines show timepoints previously analyzed (Obadan et al. 2019). Quails that were euthanize for tissue collection are marked with asterixis. † = virus was below limit of detection in the last days of experiment or data did not meet quality cut off.

Previously, we analyzed virus present in inoculated quails at 3 days post-infection (dpi) and found that L216 was positively selected when included in the inoculum (Obadan et al. 2019). Here, we sought to understand the dynamics of the selective process by analyzing the relative frequencies of the molecular markers at HA 216. Across the inoculated groups, the predominant variants included L216, Q216, M216, I216, and F216, suggesting higher relative fitness over others included in the inoculum. Furthermore, these analyses indicated that L216 reached the highest frequencies at 3 dpi in five/six quails in both groups for which L216 was included in the inoculum, var + LQ, and var + L (var + LQ: frequency = 0.47–0.96, median = 0.92; var + L: frequency = 0.42–0.98, median = 0.88), suggesting that in most animals, selection for L216 occurred at or before 3 dpi (Fig. 1 and Supplementary Table S2). L216 was also detected in five/six inoculated quails in groups for which this variant was not intentionally included in the inoculum, var∆LQ, and var + Q. Targeted NGS showed the presence of this variant at very low frequency in these inoculum (var∆LQ = 0.002; var + Q = 0.003; var + L = 0.014; var + LQ = 0.013). However, the observed L216 frequencies in the inoculum differed significantly (Kruskal–Wallis chi-squared = 9.2564, *P*-value = 0.026) (Supplementary Fig. S3 and Supplementary Table S3). The HA L216 showed distinct trajectories in the var∆LQ and var + Q groups. In the var + Q group, L216 reached consensus frequencies in five/six quails at two consecutive timepoints, whereas, in the var∆LQ group, L216 was detected at sub-consensus frequencies throughout the infection (< 0.5). These data suggest that when L216 starts the infection below certain frequency and competitors show relatively high fitness, L216 fails to reach fixation. Overall, these results show that within-host dynamics of IAV in quail receiving mixed populations of HA 216 variants was strongly shaped by positive selection and that selection can occur within the first days of the infection. In our study, this process is dependent on the initial frequency of L216 variant and the relative fitness of the competitors.

### 3.3 Presence of L216 lowers the stringency of the transmission bottleneck

To evaluate the impact of transmission on diversity at HA 216, we analyzed the relative frequency of molecular markers at this position in transmission recipients 24 h after their exposure to inoculated quails (Fig. 1). These analyses revealed that ten/twenty-four quails presented a single molecular marker at 1 dpi, while fourteen/twenty-four had at least two molecular signatures, showing that transmission often allowed for transfer of more than one HA 216 variant. Adding either the Q216 (var + Q group) or the L216 (var + L group) variant or both (var + LQ) to the mixture revealed an advantage of these variants for transmission.

The variants L216, Q216, I216, M216, and F216 were the most detected at high frequencies during transmission. Still, some minor variants present at 1 dpc reached high frequencies later in the infection. For instance, the N216 variant, which was at low frequencies in inoculated quails, was present above background in c-qa7, c-qa9, and c-qa12 at various times post-contact and it was clearly the predominant variant in c-qa11. However, the fact that L216, Q216, I216, M216, and F216 were predominant both before and after transmission suggests that positive selection has a major role in defining the transmitted virus populations.

**Figure 2.**
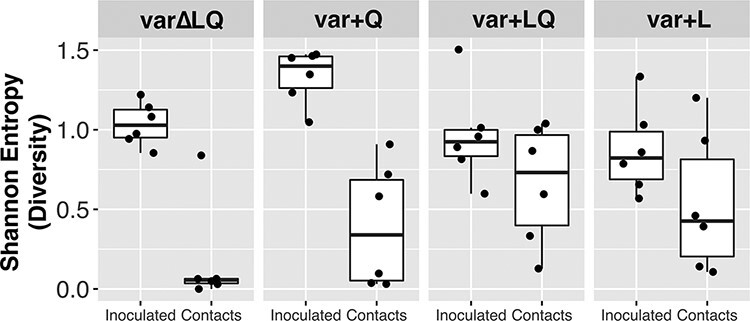
Intra-group presence of L216 at high frequencies relaxes transmission bottleneck. Shannon entropy was calculated (*y*-axis) as a measure of diversity for position HA 216. Each dot shows the mean Shannon entropy calculated using 1 dpi and 3 dpi for the inoculated quail. For the contact quails, dots represent the Shannon entropy calculation at 1 dpc. Boxplots delimit lower and upper quartile with central line showing the median.

Transmission bottlenecks determine the diversity of viral populations seeded into a new host and are therefore a critical determinant of viral adaptive potential. To quantify the effects of the transmission bottleneck on diversity at HA 216, we calculated Shannon entropy for this position. To estimate the diversity present in inoculated quails at the time of transmission (approximately 2 dpi), we calculated the mean diversity between 1 dpi and 3 dpi. The extent to which diversity was lost at transmission was then assessed by comparing the diversity in inoculated animals to that observed in contact quails at 1 dpc. The analysis revealed no significant reduction in diversity in var + L and var + LQ. Conversely, var∆LQ and var + Q showed a marked reduction (Wilcoxon test. var∆LQ: *W* = 36, *P*-value = 0.002; var + Q: *W* = 36, *P*-value = 0.002) (Fig. 2). The data therefore suggest that the intra-group presence of L216 at high frequencies allows for maintenance of diversity at the HA 216 site during transmission between hosts.

### 3.4 Selection is apparent in other viral gene segments

**Figure 3.**
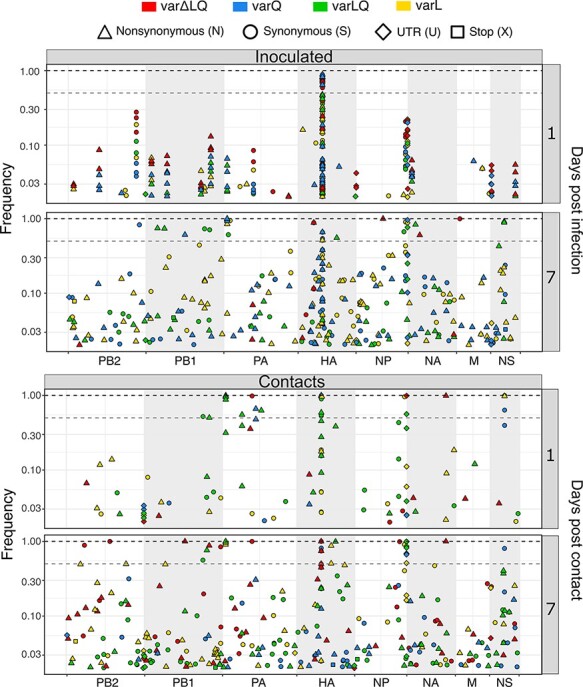
Variants distribution across influenza A genome. Variants representing different type of mutations were found to increase as the infection progressed. Days 1 and 7 are shown for inoculated and contact groups. Days post-infection are shown in the right *y*-axis. Competition groups are color coded as varΔLQ in red, var + Q in blue, var + LQ in green, and var + L in yellow. The types of mutation are represented by shapes: synonymous (S) as circles, nonsynonymous (N) as tringles, variants in the untranslated regions (U) as diamonds, and stop codons (X) as squares. In the left *y*-axis the frequency is represented in log scale from 0 to 1. The segments are shown as concatenated in the *x*-axis. Dashed grey line shows consensus cut off at 0.5 of frequency whereas dashed black line marks 1 of frequency.

Detection of the same variant in longitudinal samples from the same individual allows for evaluation of the evolutionary mechanisms that shape the genetic makeup of the viral population. This is further strengthened if the same variant is also found across individuals. To extend these analyses beyond HA 216, variant analysis across the full viral genome was performed on samples collected from the three inoculated and three contact quails that were sampled throughout the experiment (Fig. 3, Supplementary Figs S4 and S5). Synonymous, nonsynonymous and nonsense mutations were detected, as were mutations in untranslated regions of the viral genome. Most of these variants were present at a frequency between 0.02 and 0.1 throughout the infection (Supplementary Fig. S6). In general, the number of variants in inoculated and contact quails increased as the infection progressed, with an overall higher total count of nonsynonymous mutations compared to synonymous mutations (Supplementary Fig. S7). We also observed fixed or near fixed variants in contact quails by 1 dpc irrespective of the group (e.g. PB1 a1949c (N642T) and PA a891g) (Supplementary Table S4). Most of these were maintained at high frequencies (i.e. PA a100g (K26E)) throughout the infection whereas a minority, such as HA t750c and NS t436c in c-qa48, were lost as the infection progressed. These last data underline the inherent stochasticity in transmission between quails.

**Figure 4.**
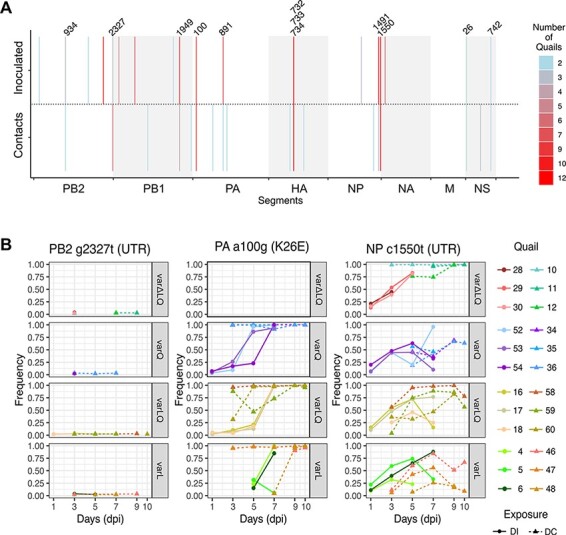
Common variants across competition and exposure groups show similar dynamics. (A) Variants were detected across quails from different competition and exposure groups. Numbers point to variants that were shared by inoculated and contact animals. Number of quails in which the variant was detected is represented in the heatmap. (B) The frequency dynamics show that some variants are kept at low frequency throughout the infection (PB2 g2327t), can be rapidly fixed (PA a100g (K26E)) or be kept from mid frequency to near fixation (NP c1550t). Direct inoculated quails are shown as dots in solid lines whereas contacts are shown as tringles in dotted lines. Days in the *x*-axis are shown as days post-infection (dpi). Each box represents the different competition groups.

The presence of the same variant across animals is a strong indicator of a selective process (Renzette et al. 2013; Parameswaran et al. 2017). Therefore, we assessed frequency dynamics of the common variants found in inoculated and contact quails to evaluate whether they were under similar selective pressures. Nine variants were identified in common between inoculated and contact quails (Fig. 4, Supplementary Fig. S8 and Table 1). A variant in the PB2 UTR, g2327t, was consistently found at low frequency on at least one day in twenty-three out of twenty-four analyzed quails (mean frequency = 0.026, SD = 0.005, range = 0.020–0.039). Failure of this variant to increase in frequency suggests a high fitness cost. In contrast, c1550t in the NP UTR was detected in all twenty-four quails and showed frequencies fluctuating from low to fixation (mean frequency = 0.516, SD = 0.304, range = 0.038–0.997). As previously inferred (Obadan et al. 2019), the PA a100g (K26E) mutation was present in quails from all groups except in var∆LQ. In the inoculated birds of the var + Q and var + LQ groups, the PA a100g (K26E) mutation was readily detected by 1 dpi (mean frequency = 0.034, SD = 0.013, range = 0.024–0.043) and all contacts from these groups carried this mutation at high frequency throughout the infection (mean frequency = 0.955, SD = 0.067, range = 0.877–0.995) (Fig. 4). To exclude that fixation of PA K26E was driven by a segment-linkage association with HA L216, we analyzed the dynamics details of both variants (Supplementary Fig. S9). As expected for advantageous variants, these two markers showed similar dynamics in some quails; however, in most birds the frequencies of L216 and E26 over time were clearly discordant, strongly suggesting that segment linkage was disrupted through reassortment. To assess whether the detected common variants arose de novo within the quail or during preparation of the virus stocks, we performed whole genome sequencing of the HA 216 variant viruses that were part of the inoculum mixtures. We found that all nine common variants were present in at least one of the twelve viruses (Supplementary Fig. S10 and Supplementary Table S5). This revealed that common variants found in inoculated and contact quails were acquired during the in vitro virus preparation. Overall, these data show that, even though within- and between-host evolution is subject to stochasticity, selective processes are important determinants shaping within- and between-host virus populations.

**Table 1.** Common variants among inoculated and direct contact groups. Nine variants were identified in common among inoculated and contact quails: PB2 a934g (T303A) and g2327t; PB1 a1949c (N642T); PA a100g (K26E), a891g; NP t1491c, c1550t; NS a26g, and g742a (NEP, E67K). Nonsynonymous mutations are shown between brackets.

EXPOSURE	PB2	PB1	PA	HA^b^	NP	NA	M1	M2	NS1	NEP
IINOCULATED	g164c (R46T)	t165g (H47Q)	a100g (K26E)		c981t				a26g (1M)^a^^,^^c^	g742a (E67K)
	a934g (T303A)	a634g (T204A)	a891g		t1491c	g118a (M33I)				
	a1604g (K527R)	a1766c/g (E581A/G)		*bld*	c1550t^a^		*bld*	*bld*		
	g2049t	a1949c (N642T)								
	g2327t^a^									
CONTACTS	a934g (T303A)	t1014a	a100g (K26E)	a625g (E190V/G)	c1344t				a26g (−1M)^a^	g742a (E67K)
	g2327t^a^	a1949c (N642T)	g589a (D189N)	g1032t (A325S)	t1491c	*bld*	*bld*	*bld*	t437c	
		a2292g	a891g		c1550t^a^					
			g1003a (E327K)							

^a^Shows variant sites at UTRs.

b None 226 sites in HA.

c Mutation introduced new start codon at −1 position from canonical start codon. The legend *bld* corresponds to below limit of detection.

### 3.5 Presence of HA L216 allowed for greater whole genome diversity after transmission

**Figure 5.**
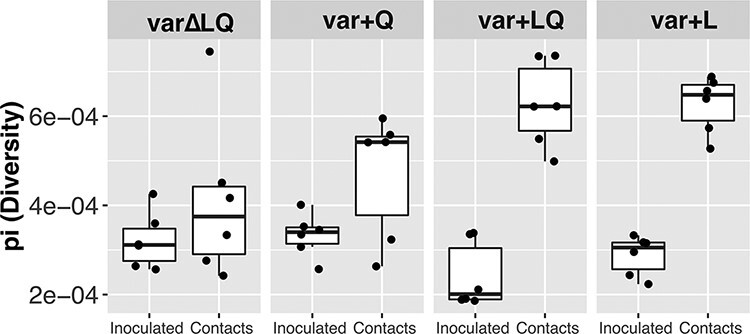
Inclusion of HA L216 allows for a greater diversification at the whole genome level. To assess diversity at the genome level, π was calculated. Each dot shows the mean π calculated using 1 dpi and 3 dpi for the inoculated quails representing the time point at which the naïve quails were introduced—2dpi. For the contact quails, dots represent the π calculation at 1 dpc. Central line in box plots shown the median.

We then compared diversity at the whole genome level before and after transmission. We calculated π as a diversity metric, excluding the position HA 216 from the analysis. As in the analysis for HA 216, we calculated the mean diversity between 1 and 3 dpi in the inoculated quails and compared the results with the diversity detected at 1 dpc in the contact birds (Fig. 5). The results show that the transmission event did not reduce viral genetic diversity. Differences in virus diversity before and after transmission in var∆LQ and var + Q were not significant (Wilcoxon test. var∆LQ: W = 13, *P*-value = 0.484; var + Q: W = 9, *P*-value = 0.179). In contrast, virus populations from the var + LQ and var + L groups showed significantly greater diversity at 1 dpc in the contact quails compared to the inoculated quails (Wilcoxon test. var + LQ: W = 0, *P*-value = 0.002; var + L: W = 0, *P*-value = 0.002). This increase in diversity may reflect either a more efficient transfer of variants from the donor(s), an increase in production of new intra-host variants or a mix of both. Overall, these data suggest that inclusion of HA L216 in the inoculum mixture was associated with greater diversity at 1 dpc.

## 4. Discussion

The extent to which selective and stochastic processes shape influenza virus populations infecting poultry are poorly understood. In the present report we analyzed time series data—obtained through longitudinal sampling—from a multi-competitive infection in quail, a natural host of poultry-adapted H9N2 IAVs. Contrary to what has been described in humans (Debbink et al. 2017; McCrone et al. 2018; Valesano et al. 2020), we found that selective processes shaped intra- and inter-host virus populations. However, we also found that selection of HA L216 depended on the competition context. Surprisingly, the presence of the HA L216 variant enabled greater diversity at transmission. Although our previous study showed the biological advantage of the HA L216 variant for replication and transmission in quail, questions remained regarding the dynamics of the selective processes and the interplay with other major and/or minor variants elsewhere in the genome. Our current analyses show that when the L216 variant meets a minimal frequency in the inoculum, it is quickly fixed in most inoculated birds. However, virus population dynamics varied at the host level allowing alternative variants such as I216 and M216 to alternate at different times post-inoculation, clearly showing that alternative variants possess enough fitness to compete with the canonical molecular markers.

The fate of beneficial mutations depends on their fitness advantage, their frequency, and time of introduction (Gerrish and Lenski 1998; Miralles et al. 1999). Hence, we propose that the very low initial frequencies of L216 in the var∆LQ and var + Q groups, together with the relatively high fitness of other variants and a large founding population size, resulted in variant dynamics consistent with clonal interference (Gerrish and Lenski 1998; Miralles et al. 1999; Kao and Sherlock 2008; Lang et al. 2013). In large populations, natural selection operates by selecting the most fit variant and eventually leading to its fixation. However, in viruses and other microorganisms, fixation of advantageous variants can be disrupted (Gerrish and Lenski 1998; Kao and Sherlock 2008; Lang et al. 2013). This model explains that fixation of beneficial variants decreases when competitors show similar fitness and the population size is large (Gerrish and Lenski 1998; Miralles, Moya, and Elena 2000). These two features are present in our experiment. The founding population in the inoculated animals was rather large (10^6^ TCID_50_/quail) and the comparable fitness of some of the alternative variants—such as M, F, and I—can explain the failure of L216 to fix in the inoculated quails from the var∆LQ group. Additionally, above-consensus frequencies of L216 in the var + Q were transient. Although transient high frequencies of beneficial variants is an established feature of population dynamics dominated by clonal interference (Gerrish and Lenski 1998; Hughes et al. 2012; Lang et al. 2013; Maddamsetti, Lenski, and Barrick 2015), this observation further highlights the complexity of the multiple competition at HA 216. How the interplay between the HA 216 variants that are rare in nature—I, M, and F—and the canonical markers modulates competition dynamics warrants further research. Based on these results, we speculate that clonal interference can interrupt adaptation of influenza virus SA recognition, consistent with molecular epidemiological studies of IAVs in humans (Strelkowa and Lassig 2012).

Variants such as PB2 g2327t, PA a100g (K26E), and NP t1550c showed similar dynamics across multiple individuals in inoculated and contact groups, suggesting that these variants are shaped by the same selective pressures among quails, independent of the HA 216 competition regime. The frequencies of these three variants—and those of HA 216 markers—furthermore showed clear discrepancies within a given quail. These observations can be explained in a context where reassortment is efficient enough to allow for segment-independent evolution (Marshall et al. 2013; White and Lowen 2018). This scenario is likely to occur when cellular coinfection is common and segment mismatch is minor or absent (White and Lowen 2018). Prior work has established that IAV coinfection occurs frequently in vivo (Brooke et al. 2014; Phipps et al. 2020; Ganti et al. 2021). Even though the mixture of viruses were diverse in HA, the remaining segments were alike, likely placing minimal constraints on reassortment and therefore enabling segment-independent evolution.

The transmission bottleneck describes the reduction of the virus populations transferred from donor to recipient. Bottlenecks are important because, for rapidly evolving pathogens such as influenza virus, narrow bottlenecks reduce the diversity transferred and thereby reduce adaptive potential. Viral factors modulating the stringency of IAV transmission bottlenecks are not well characterized. Our analysis showed that the presence of L216 in infecting virus populations allowed the transfer of a greater diversity of HA 216 genotypes to recipient birds. We speculate that this phenomenon can be explained by a mechanism of phenotypic hiding (Wilke and Novella 2003), in which a virus particle carrying HA L216 protein on its surface encodes other molecular markers in its genome. Such viral pseudotyping can occur when multiple variant viruses coinfect the same cell, as has been seen to commonly occur in vivo (Brooke et al. 2014; Phipps et al. 2020; Ganti et al. 2021). The access of viruses decorated with HA L216 to cells containing more α2-6SA, which are abundant in the quail respiratory tract, may have contributed to efficient coinfection in inoculated hosts and transmission to recipient hosts. Under this model, the coupling of the HA L216 phenotype with diverse HA 216 genotypes allowed for initial propagation of a diverse population. Then, over time, L216 dominated due to its higher fitness and some degree of spread in the absence of coinfection. Importantly, a similar trend of higher diversity after transmission was observed at the whole genome level when L216 was included, which would be expected to facilitate selection. Overall, the inclusion of HA L216 appeared to confer advantages to the virus populations.

Donor quails were inoculated through nares, trachea, and cloaca. Even though the most frequent SA conformation in the nares and trachea is α 2-6, SA in the α 2-3 conformation is also found, for instance, in the goblet cells of the respiratory tract (Wan and Perez 2006; Yamada et al. 2012). This dual receptor availability explains the capacity of viruses with the ability to recognize α 2-3, α 2-6, or both SA conformations to infect the respiratory tract of quails. This feature is a major reason why this species may act as an intermediate host between wild birds and mammals (Thontiravong et al. 2012). In our experimental system, individual variability in expression of each receptor, may have shaped the competition of virus populations in the trachea. Virus was not produced at detectable levels in the gastrointestinal tract, consistent with the respiratory tropism IAVs in poultry (Wan and Perez 2006). This tropism suggests that the transmission route was respiratory and may have involved inhaled aerosols, droplet spray and/or direct contact. The contact structure of quails used in our experiments resembles that used in the poultry industry where animals are in direct contact. Prior work in mammalian models indicates that transmission among animals in direct contact is characterized by a looser bottleneck compared to transmission where animals are physically separated (Varble et al. 2014; Frise et al. 2016). The high population density of poultry farmed commercially may therefore support transmission of larger numbers of viral variants. Indeed, we propose that the resultant high viral population diversity within a group of birds may promote constant exposure of individuals to novel variants. We argue that these environments can create optimal conditions for selective processes to act, resulting in rapid viral adaptation.

Overall, we show that, in avian IAV infecting a natural host, selection is able to act efficiently to fix advantageous mutations within a single host infection cycle and that selection acts at the level of transmission. Of particular note, we found that transmission bottlenecks and diversity in the recipient host can differ depending on molecular markers that modulate host recognition. Our findings shed light on the mechanisms that allow avian IAV to become fitter in their natural host and highlight population traits—such as loose genetic bottlenecks—that have the potential to permit access to new hosts.

## Supplementary Material

veac001_Supp

## Data Availability

The sequences are available through NCBI’s Short Read Archive (https://www.ncbi.nlm.nih.gov/sra) BioProject accession number PRJNA766620. All custom computer code necessary to reproduce the results presented in the manuscript are available on GitHub (https://github.com/genferreri/Intra--and-inter-host-evolution-of-H9N2-influenza-A-virus-in-Japanese-quail).
