# Supplementary material for: Intra- and inter-host evolution of H9N2 influenza A virus in Japanese quail
Source: Virus Evol. 2022 Jan 8;8(1):veac001. doi: 10.1093/ve/veac001 (PMC8865083; doi:10.1093/ve/veac001)
Supplement: veac001_Supp [file veac001_supp.zip › SuppementaryTable_4.docx]

| Quail | Segment | Position | REF | ALT | Frequency | REF-AA | ALT-AA | Mutation type | Day | Group |
| --- | --- | --- | --- | --- | --- | --- | --- | --- | --- | --- |
| 10 | 3 | 891 | A | G | 0.983 | K | K | S | 1 | var∆LQ |
| 10 | 3 | 891 | A | G | 0.995 | K | K | S | 3 | var∆LQ |
| 10 | 3 | 891 | A | G | 0.997 | K | K | S | 7 | var∆LQ |
| 10 | 3 | 891 | A | G | 0.998 | K | K | S | 5 | var∆LQ |
| 10 | 3 | 891 | A | G | 0.998 | K | K | S | 8 | var∆LQ |
| 10 | 5 | 1550 | C | T | 0.990 | NA | NA | U | 1 | var∆LQ |
| 10 | 5 | 1550 | C | T | 0.996 | NA | NA | U | 3 | var∆LQ |
| 10 | 5 | 1550 | C | T | 0.997 | NA | NA | U | 7 | var∆LQ |
| 10 | 5 | 1550 | C | T | 0.987 | NA | NA | U | 5 | var∆LQ |
| 10 | 5 | 1550 | C | T | 0.993 | NA | NA | U | 8 | var∆LQ |
| 10 | 6 | 1167 | C | T | 0.989 | T | M | N | 1 | var∆LQ |
| 10 | 6 | 1167 | C | T | 0.994 | T | M | N | 3 | var∆LQ |
| 10 | 6 | 1167 | C | T | 0.998 | T | M | N | 7 | var∆LQ |
| 10 | 6 | 1167 | C | T | 0.994 | T | M | N | 5 | var∆LQ |
| 10 | 6 | 1167 | C | T | 0.997 | T | M | N | 8 | var∆LQ |
| 34 | 3 | 100 | A | G | 0.993 | K | E | N | 1 | var+Q |
| 34 | 3 | 100 | A | G | 0.993 | K | E | N | 3 | var+Q |
| 34 | 3 | 100 | A | G | 0.997 | K | E | N | 5 | var+Q |
| 34 | 3 | 100 | A | G | 0.998 | K | E | N | 7 | var+Q |
| 34 | 3 | 100 | A | G | 0.996 | K | E | N | 8 | var+Q |
| 35 | 3 | 100 | A | G | 0.997 | K | E | N | 1 | var+Q |
| 35 | 3 | 100 | A | G | 0.997 | K | E | N | 3 | var+Q |
| 35 | 3 | 100 | A | G | 0.994 | K | E | N | 5 | var+Q |
| 36 | 3 | 100 | A | G | 0.996 | K | E | N | 1 | var+Q |
| 36 | 3 | 100 | A | G | 0.989 | K | E | N | 3 | var+Q |
| 36 | 3 | 100 | A | G | 0.910 | K | E | N | 5 | var+Q |
| 36 | 3 | 100 | A | G | 0.998 | K | E | N | 7 | var+Q |
| 36 | 3 | 100 | A | G | 0.998 | K | E | N | 8 | var+Q |
| 36 | 8 | 437 | T | C | 0.997 | I | I | S | 1 | var+Q |
| 36 | 8 | 437 | T | C | 0.832 | I | I | S | 3 | var+Q |
| 36 | 8 | 437 | T | C | 0.878 | I | I | S | 5 | var+Q |
| 36 | 8 | 437 | T | C | 0.804 | I | I | S | 7 | var+Q |
| 36 | 8 | 437 | T | C | 0.614 | I | I | S | 8 | var+Q |
| 47 | 3 | 100 | A | G | 0.952 | K | E | N | 1 | var+L |
| 47 | 3 | 100 | A | G | 0.980 | K | E | N | 3 | var+L |
| 47 | 3 | 100 | A | G | 0.969 | K | E | N | 5 | var+L |
| 47 | 3 | 100 | A | G | 0.995 | K | E | N | 7 | var+L |
| 47 | 3 | 100 | A | G | 0.997 | K | E | N | 8 | var+L |
| 47 | 5 | 1491 | T | C | 0.960 | S | S | S | 1 | var+L |
| 47 | 5 | 1491 | T | C | 0.871 | S | S | S | 3 | var+L |
| 47 | 5 | 1491 | T | C | 0.839 | S | S | S | 5 | var+L |
| 47 | 5 | 1491 | T | C | 0.907 | S | S | S | 7 | var+L |
| 47 | 5 | 1491 | T | C | 0.988 | S | S | S | 8 | var+L |
| 48 | 4 | 750 | T | C | 0.965 | Y | H | N | 1 | var+L |
| 48 | 4 | 750 | T | C | 0.037 | Y | H | N | 3 | var+L |
| 48 | 4 | 750 | T | C | 0.121 | Y | H | N | 5 | var+L |
| 48 | 4 | 750 | T | C | bld | Y | H | N | 7 | var+L |
| 48 | 4 | 750 | T | C | bld | Y | H | N | 8 | var+L |
| 48 | 8 | 436 | T | C | 0.977 | I | T | N | 1 | var+L |
| 48 | 8 | 436 | T | C | 0.034 | I | T | N | 3 | var+L |
| 48 | 8 | 436 | T | C | 0.067 | I | T | N | 5 | var+L |
| 48 | 8 | 436 | T | C | bld | I | T | N | 7 | var+L |
| 48 | 8 | 436 | T | C | bld | I | T | N | 8 | var+L |
| 58 | 3 | 100 | A | G | 0.957 | K | E | N | 1 | var+LQ |
| 58 | 3 | 100 | A | G | 0.987 | K | E | N | 3 | var+LQ |
| 58 | 3 | 100 | A | G | 0.983 | K | E | N | 5 | var+LQ |
| 58 | 3 | 100 | A | G | 0.996 | K | E | N | 7 | var+LQ |
| 58 | 3 | 100 | A | G | 0.998 | K | E | N | 8 | var+LQ |
