# Supplementary material for: Intra- and inter-host evolution of H9N2 influenza A virus in Japanese quail
Source: Virus Evol. 2022 Jan 8;8(1):veac001. doi: 10.1093/ve/veac001 (PMC8865083; doi:10.1093/ve/veac001)
Supplement: veac001_Supp [file veac001_supp.zip › SupplementaryTable_5.docx]

| Virus Position HA 216 | Segment | Position | Coverage | REF | ALT | Frequency | REF-AA | ALT-AA | Mutation type |
| --- | --- | --- | --- | --- | --- | --- | --- | --- | --- |
| Iso | 3 | 1949 | 2247 | C | A | 0.038 | Ala | Glu | N |
| Iso | 5 | 1550 | 3913 | C | T | 0.030 | *NA* | *NA* | U |
| Cys | 5 | 1550 | 3019 | C | T | 0.982 | *NA* | *NA* | U |
| Gly | 1 | 2327 | 775 | G | T | 0.026 | *NA* | *NA* | U |
| Ser | 1 | 2327 | 1442 | G | T | 0.025 | *NA* | *NA* | U |
| His | 5 | 1550 | 1417 | C | T | 0.994 | *NA* | *NA* | U |
| Met | 1 | 934 | 3699 | A | G | 0.224 | Thr | Ala | N |
| Met | 1 | 2327 | 1833 | G | T | 0.034 | *NA* | *NA* | U |
| Met | 3 | 891 | 2595 | A | G | 0.206 | Lys | Lys | S |
| Met | 5 | 1491 | 5532 | T | C | 0.565 | Ser | Ser | S |
| Met | 8 | 26 | 8003 | A | G | 0.201 | *NA* | *NA* | U |
| Met | 8 | 742 | 7920 | G | A | 0.225 | Glu | Lys | N |
| Phe | 2 | 1949 | 1664 | A | C | 0.993 | Asn | Thr | N |
| Val | 1 | 2327 | 2804 | G | T | 0.027 | *NA* | *NA* | U |
| Gln | 1 | 2327 | 2423 | G | T | 0.043 | *NA* | *NA* | U |
| Gln | 3 | 100 | 2570 | A | G | 0.157 | Lys | Glu | N |
| Leu | 1 | 2327 | 4199 | G | T | 0.026 | *NA* | *NA* | U |
