# Supplementary material for: Intra- and inter-host evolution of H9N2 influenza A virus in Japanese quail
Source: Virus Evol. 2022 Jan 8;8(1):veac001. doi: 10.1093/ve/veac001 (PMC8865083; doi:10.1093/ve/veac001)
Supplement: veac001_Supp [file veac001_supp.zip › Supplementary legends.docx]

**Supplementary table 1. Primer list used for targeted Next Generation Sequencing.** All sequences are oriented 5’ to 3’.

**Supplementary figure 1. Determining workflow error and cut off for variant analysis.**

A) Primers were designed to amplify beta lactamase gene (Bla) and to produce influenza A UTR ends recognized by the OPTI primers used in the MS-RTPCR. Reverse primer also added the necessary sequence for T7 polymerase to produce RNA in vitro. The synthetic RNA was treated following the NGS workflow for Influenza A sequencing. The analysis was performed using the Bla sequence as reference. B) Variant analysis of three independent amplifications.

**Supplementary figure 2.** Viral shedding was quantified by quantitative reverse transcriptase PCR in tracheal swabs. This figure was previously published in Obadan et al., 2019.

**Supplementary table 2. Variant analysis from the in vivo competition at position HA 216 in inoculated and direct contact quails**. The table shows read count and frequency for all amino acids at position HA 216 per quail during infection. # in Quail column denotes ID. “DI” and “DC” in exposure denote direct inoculated and direct contact respectively. The “day” column shows dpi for DI and dpc for DC.

**Supplementary figure 3. Inoculum characterization using targeted NGS.** PCR products targeting HA 216 were produced to evaluate the inoculum composition used in the in vivo competition experiment. L216 was detected in var∆LQ and var+Q (frequencies of 2x10^-3^ and 3x10^-3^ respectively) and Q216 in var∆LQ and var+L (frequencies of 1.4x10^-2^ and 1.3x10^-2^ respectively) along with others low frequency variants including alanine, aspartic acid, glutamic acid, lysine, proline, arginine, tryptophan, tyrosine, and stop codons (mean frequencies ranged from 3.39x10^-6^ for D in inoculum var∆LQ to 1.69x10^-3^ for P in inoculum var+Q).

**Supplementary table 3**. HA216 target NGS performed in inoculum mixtures. Mean frequency was calculated from 3 independent sequencing replicates. The “*” represents the stop codon.

**Supplementary figure 4. Variants distribution across influenza A genome in inoculated groups.** Days post infection are shown in the right y-axis. Competition groups are color coded as varΔLQ in red, var+Q in blue, var+LQ in green and var+L in yellow. The types of mutation are represented by shapes: synonymous (S) as circles, nonsynonymous (N) as tringles, variants in the untranslated regions (U) as diamonds and stop codons (X) as squares. In the left y-axis the frequency is represented in a log scale from 0 to 1. The segments are shown as concatenated in the x-axis. Dashed grey line shows consensus cut off at 0.5 of frequency whereas dashed black line delimits frequency of 1.

**Supplementary figure 5. Variants distribution across influenza A genome in direct contact groups.** Days post infection are shown in the right y-axis. Competition groups are color coded as varΔLQ in red, var+Q in blue, var+LQ in green and var+L in yellow. The types of mutation are represented by shapes: synonymous (S) as circles, nonsynonymous (N) as tringles, variants in the untranslated regions (U) as diamonds and stop codons (X) as squares. In the left y-axis the frequency is represented in a log scale from 0 to 1. The segments are shown as concatenated in the x-axis. Dashed grey line shows consensus cut off at 0.5 of frequency whereas dashed black line delimits frequency of 1.

**Supplementary figure 6. Most variants are present at low frequency throughout the infection.** Mutation type and relative frequencies at different time points in the infection are shown. Proportions are depicted in the y-axis divided into exposure groups. Range of frequencies are shown in the x-axis. Days are represented by dpi or dpc for inoculated and contacts respectively. Types of mutations are named as N for nonsynonymous, S for synonymous, U for mutations present in the UTRs and X for nonsense.

**Supplementary figure 7. Consistent increment of total number of variants after 3 dpi/dpc.** Days are shown as number of dpi or dpc for inoculated and contacts respectively. Aggregated data for types of mutations are showed as box and whisker plots. Types of mutations are named as N for nonsynonymous, S for synonymous, U for mutations present in the UTRs and X for nonsense.

**Supplementary table 4. Transmission by direct contact promotes moderate fixation of variants.** Only contact quails are shown in the table. Frequency values close to 1 at 1 dpc strongly suggest fixation upon transmission. REF refers to nucleotide in the reference sequence. ALT refers to the alternative variant found. REF-AA and ALT-AA refer to the amino acid encoded by the reference nucleotide and its alternative respectively. Nucleotide changes in the untranslated regions are denoted as *NA* in these last two columns. Types of mutations are named as N for nonsynonymous, S for synonymous, U for mutations present in the UTRs. “*bld*” = below limit of detection.

**Supplementary figure 8. Evolutionary dynamics for common variants.** Frequency trajectory of variants shared across quails. Direct inoculated quails are shown as dots in solid lines whereas contacts are shown as tringles in dotted lines. Days in the x-axis are shown as days post infection (dpi). Each box represents the different competition groups.

**Supplementary figure 9. Frequency dynamics comparison between HA L216 and PA K26E suggests lack of segment linkage between HA and PA.** Only quails in which PA 26E was detected more than once are shown.

**Supplementary figure 10. Variants found in the in vivo experiment were detected in the viruses used to produce the inoculums.** Individual viruses used for inoculum mixture were sequenced by NGS. The different colors depict each HA 216 variant virus. Types of mutations are named as N for nonsynonymous (triangles), S for synonymous (circles), U for mutations present in the UTRs (diamonds). In the left y-axis the frequency is represented in a log scale from 0 to 1. The segments are shown as concatenated in the x-axis. Dotted grey line shows consensus cut off at 0.5 of frequency whereas dotted black line marks frequency of 1.

**Supplementary table 5. Common variants found in the in vivo quail experiment were detected on individual reverse genetic viruses used to generate the inoculums.** Each of the viruses used to generate the inoculum mixes were sequenced by NGS. The first column shows the virus with the amino acid at position HA 216. Different virus rescues shared some of these variants. REF refers to nucleotide in the reference sequence. ALT refers to the alternative variant found. REF-AA and ALT-AA refer to the amino acid encoded by the reference nucleotide and its alternative respectively. Nucleotide changes in the untranslated regions are denoted as *NA* in these last two columns. Types of mutations are named as N for nonsynonymous, S for synonymous, U for mutations present in the UTRs.
