# Supplementary figures and images for: Intra- and inter-host evolution of H9N2 influenza A virus in Japanese quail
Source: Virus Evol. 2022 Jan 8;8(1):veac001. doi: 10.1093/ve/veac001 (PMC8865083; doi:10.1093/ve/veac001)

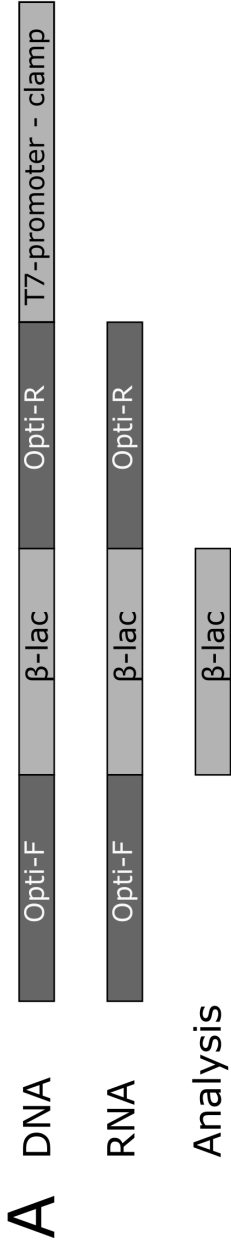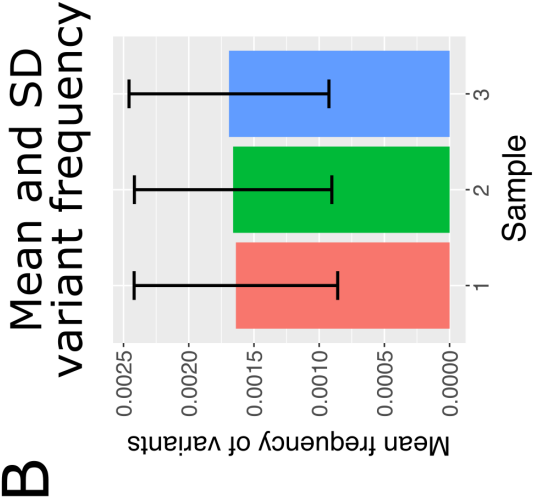

Supplement: veac001_Supp [file veac001_supp.zip › SuppFigure 1.pdf]

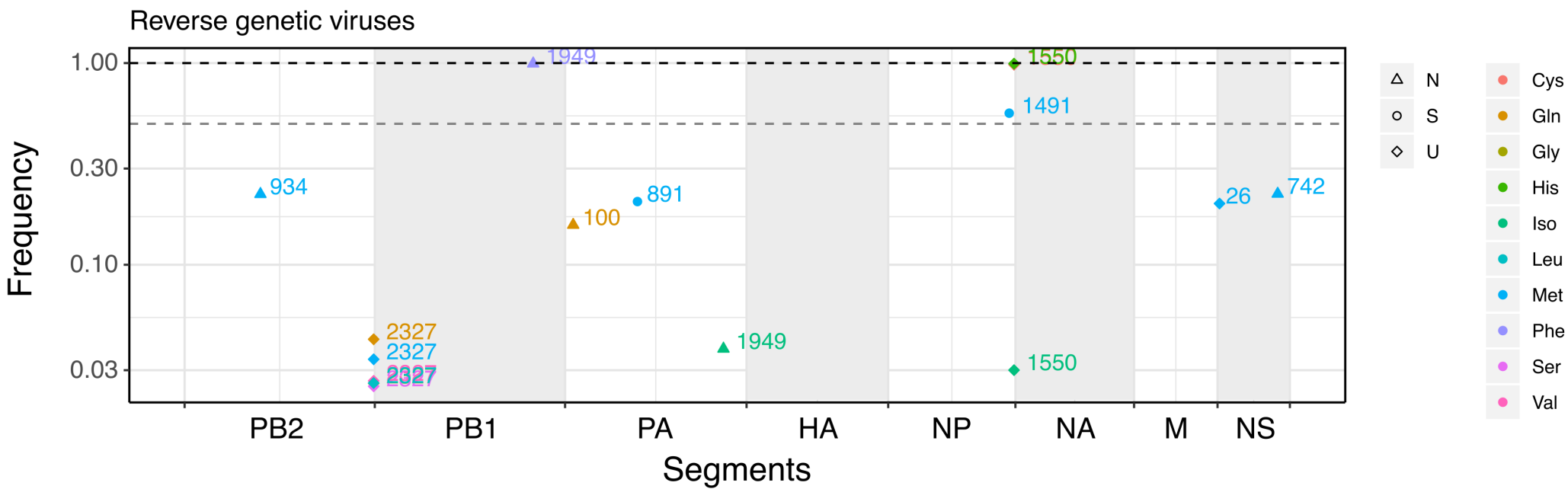

Supplement: veac001_Supp [file veac001_supp.zip › SuppFigure 10 .pdf]

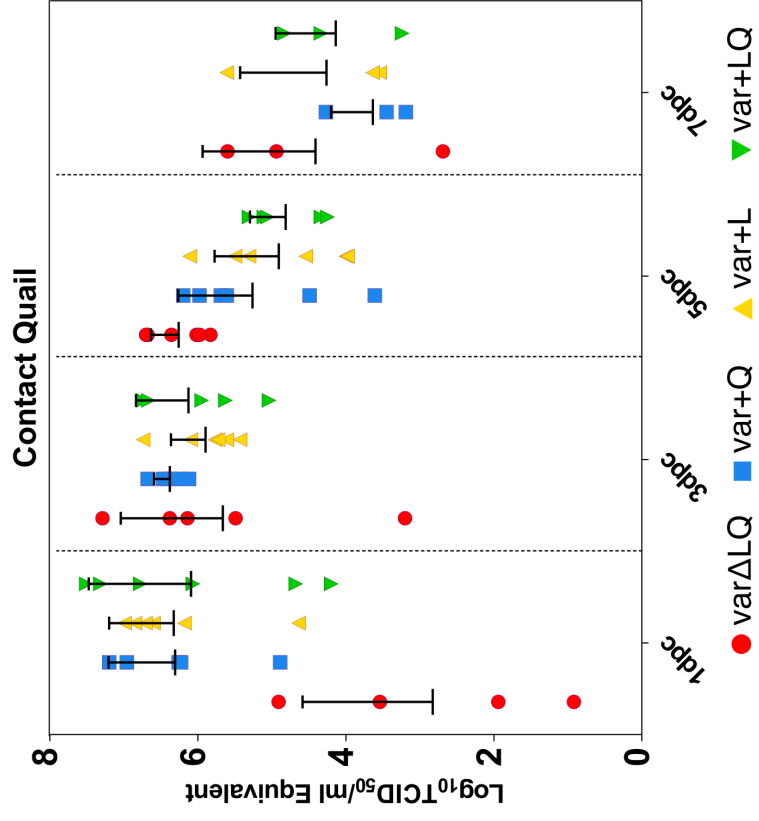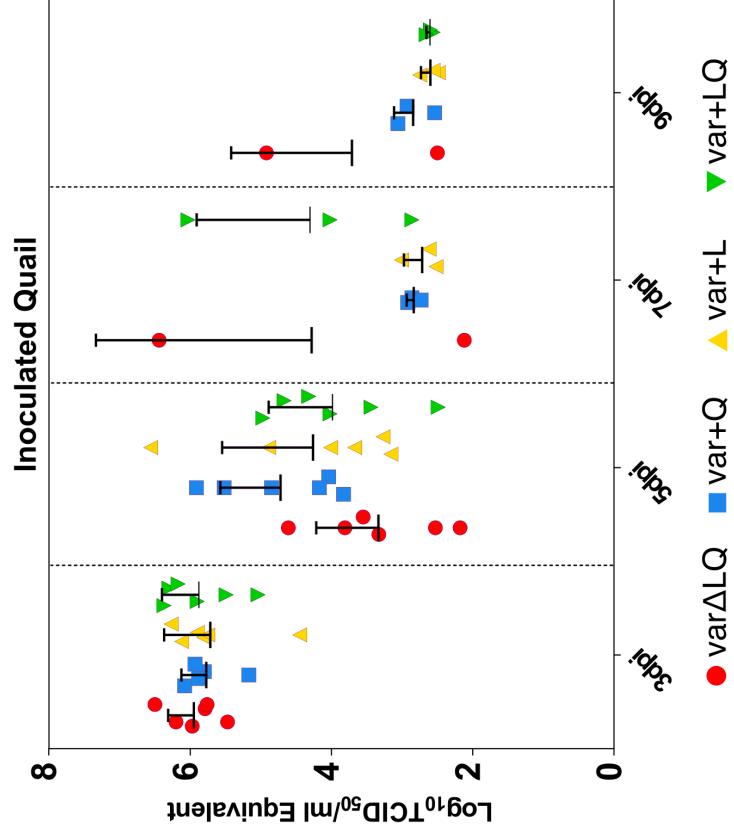

Supplement: veac001_Supp [file veac001_supp.zip › SuppFigure 2.pdf]

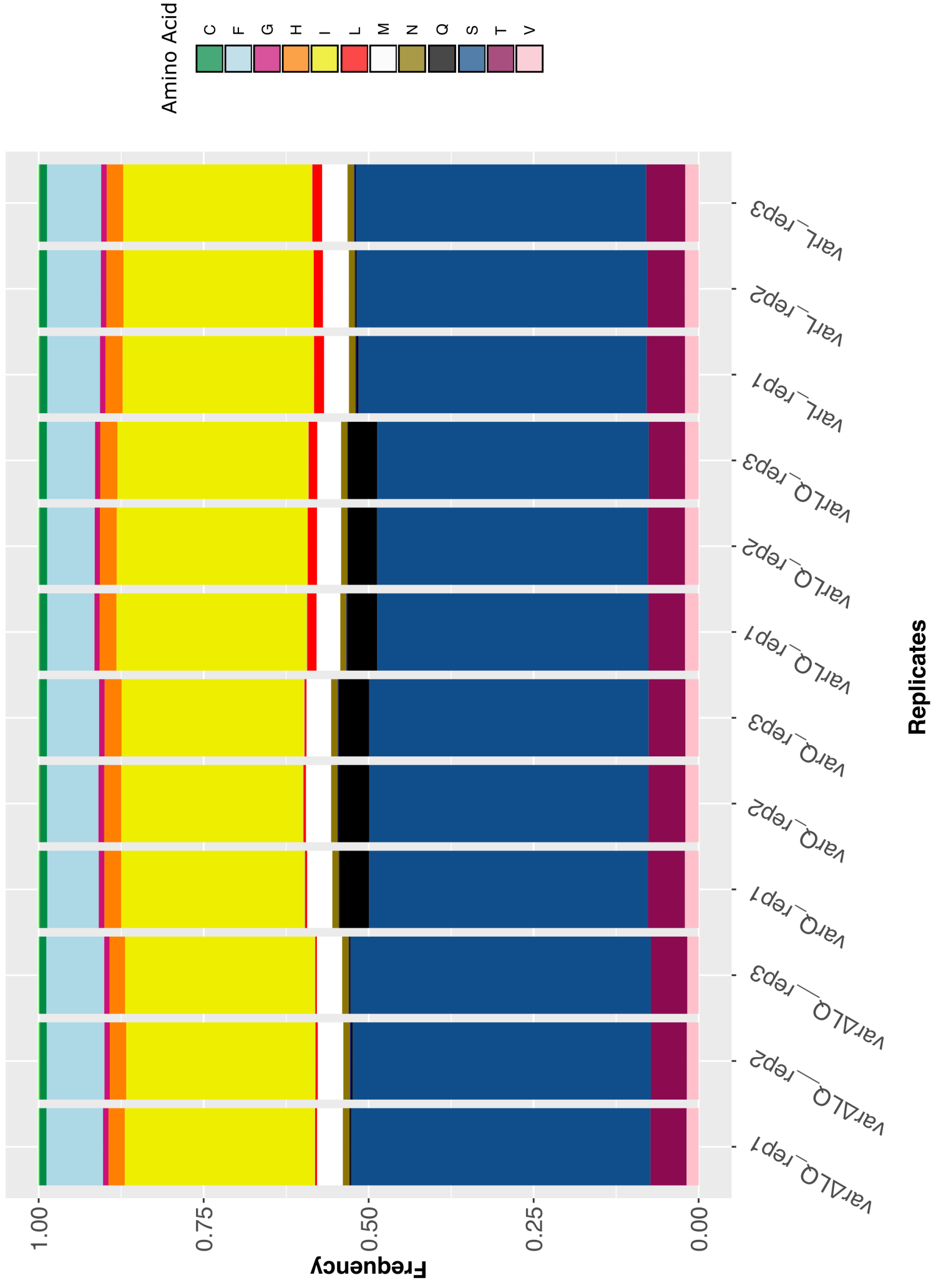

Supplement: veac001_Supp [file veac001_supp.zip › SuppFigure 3.pdf]

■ var $\Delta$ LQ   
 ■ varL   
 ■ varLQ   
 ■ varQ  
△ Nonsynonymous (N)   
○ Synonymous (S)   
◇ UTR (U)   
□ Stop (X)

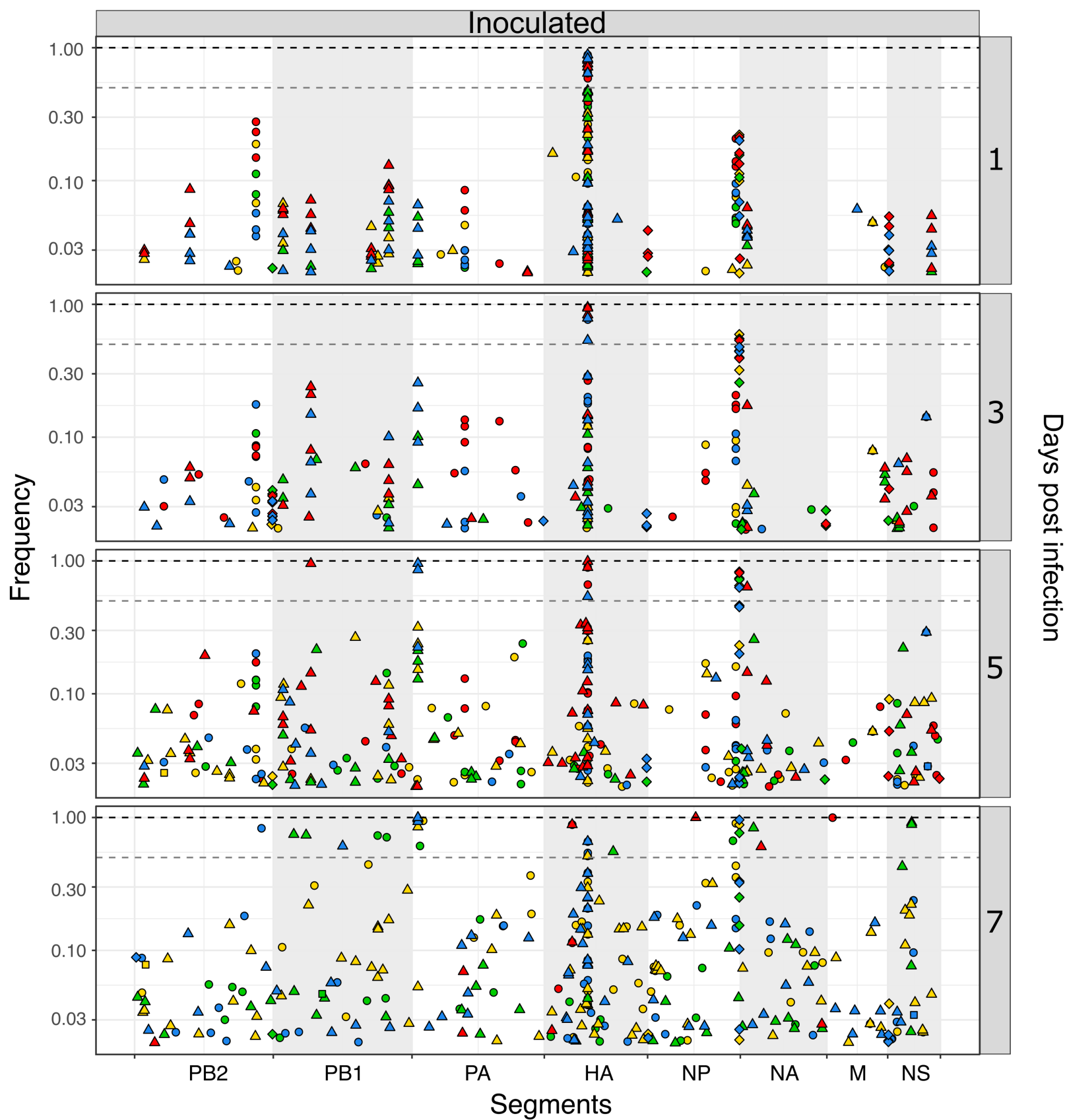

Supplement: veac001_Supp [file veac001_supp.zip › SuppFigure 4.pdf]

■ var $\Delta$ LQ    ■ varL    ■ varLQ    ■ varQ  
△ Nonsynonymous (N)    ○ Synonymous (S)    ◇ UTR (U)    □ Stop (X)

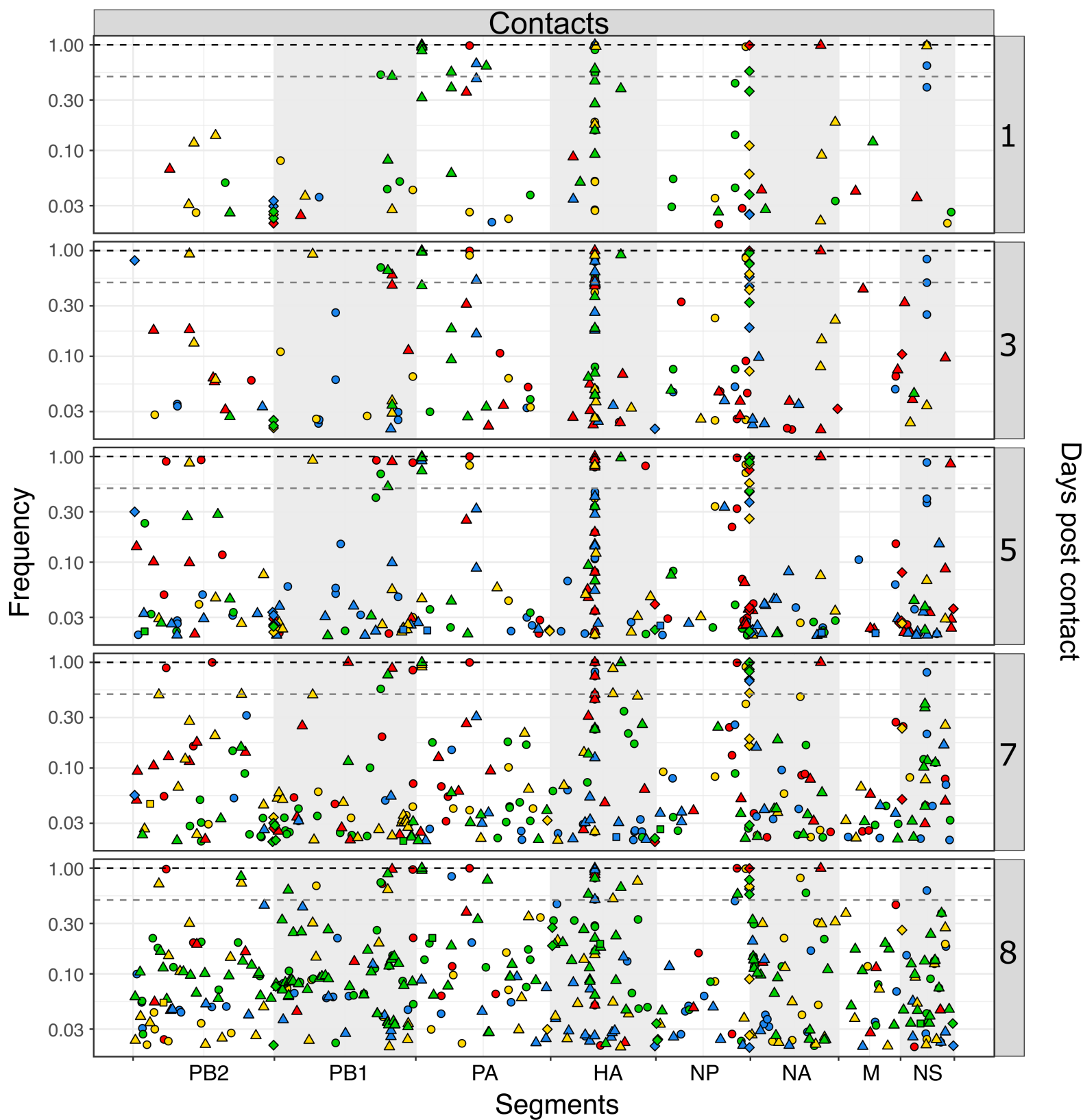

Supplement: veac001_Supp [file veac001_supp.zip › SuppFigure 5.pdf]

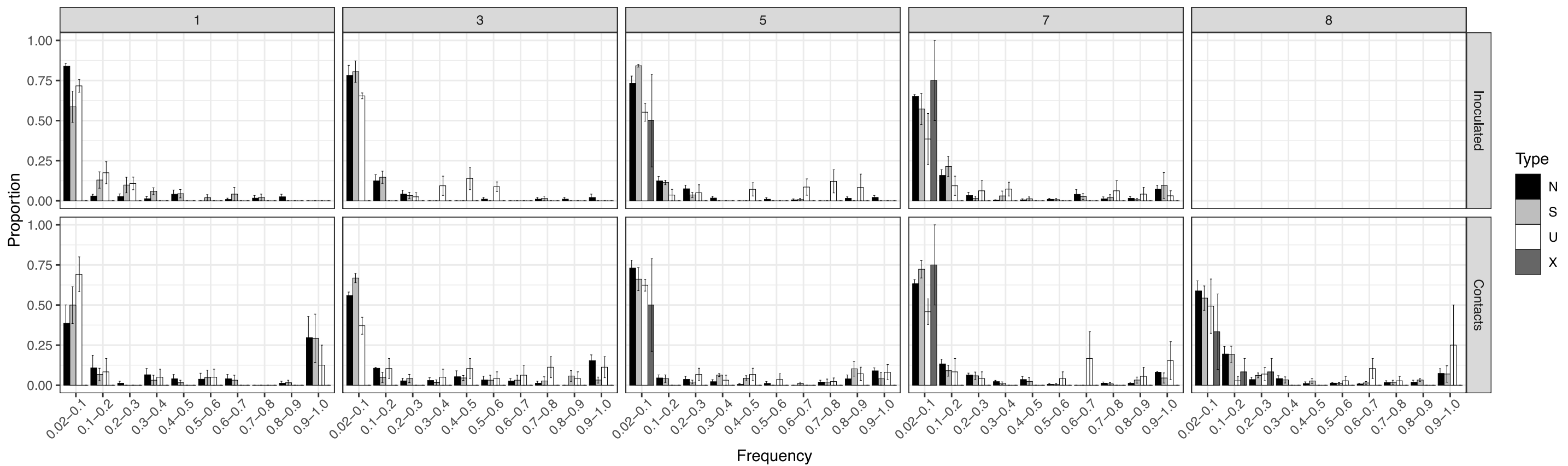

Supplement: veac001_Supp [file veac001_supp.zip › SuppFigure 6 .pdf]

Inoculated

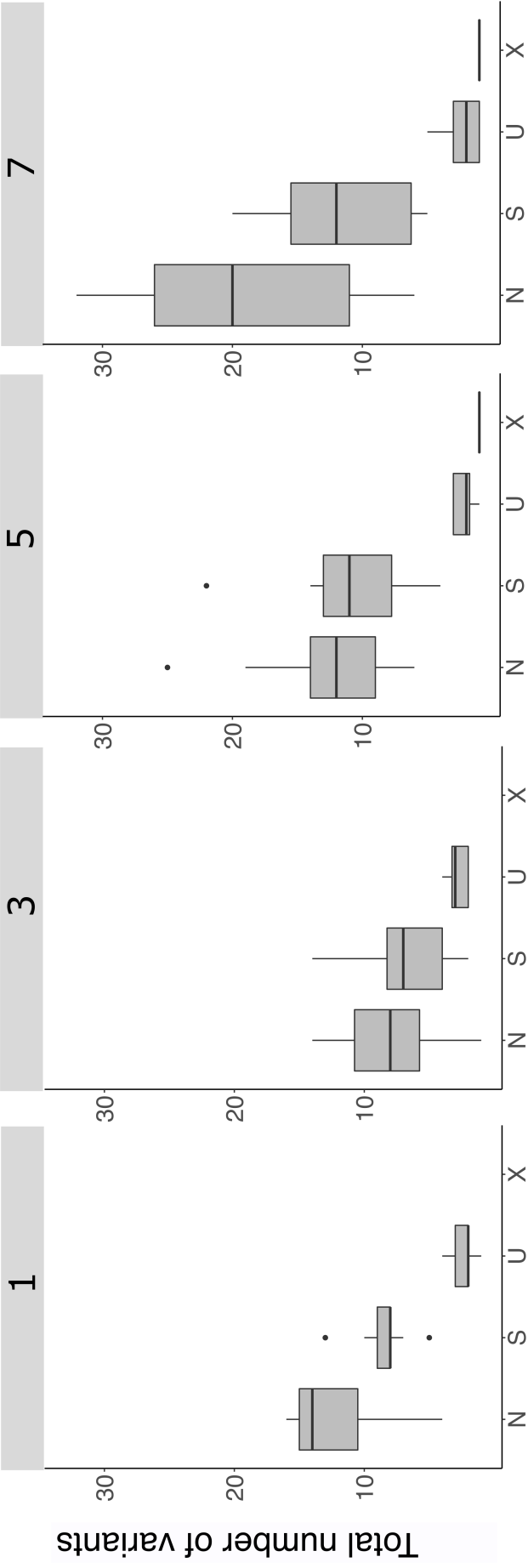

Contacts

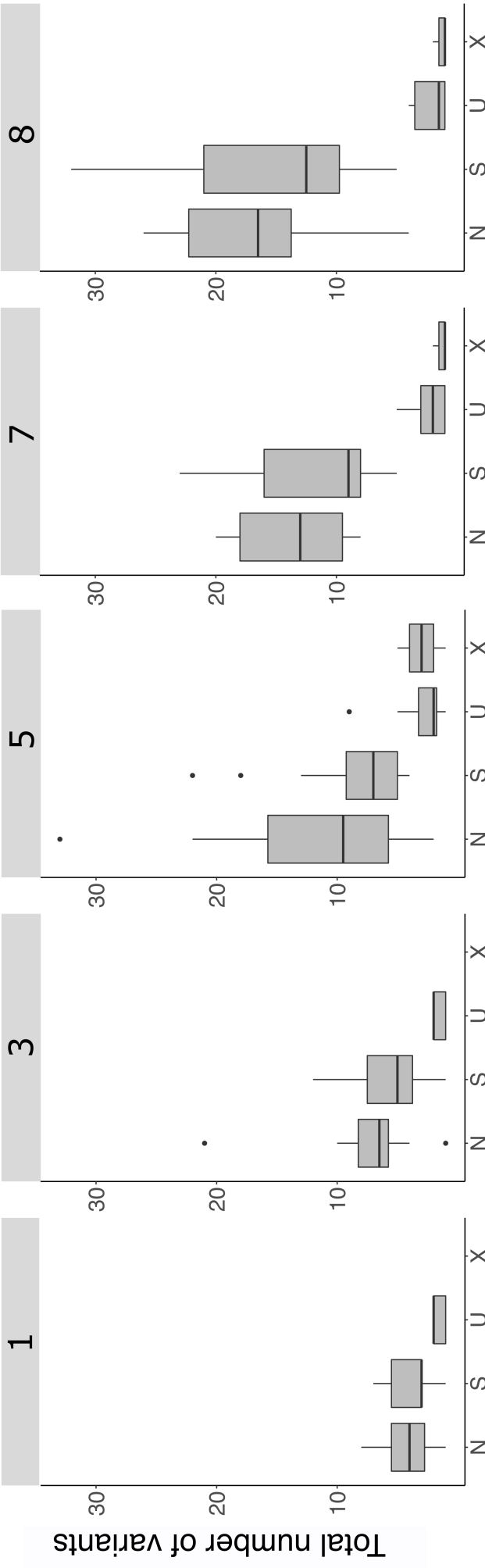

Supplement: veac001_Supp [file veac001_supp.zip › SuppFigure 7.pdf]

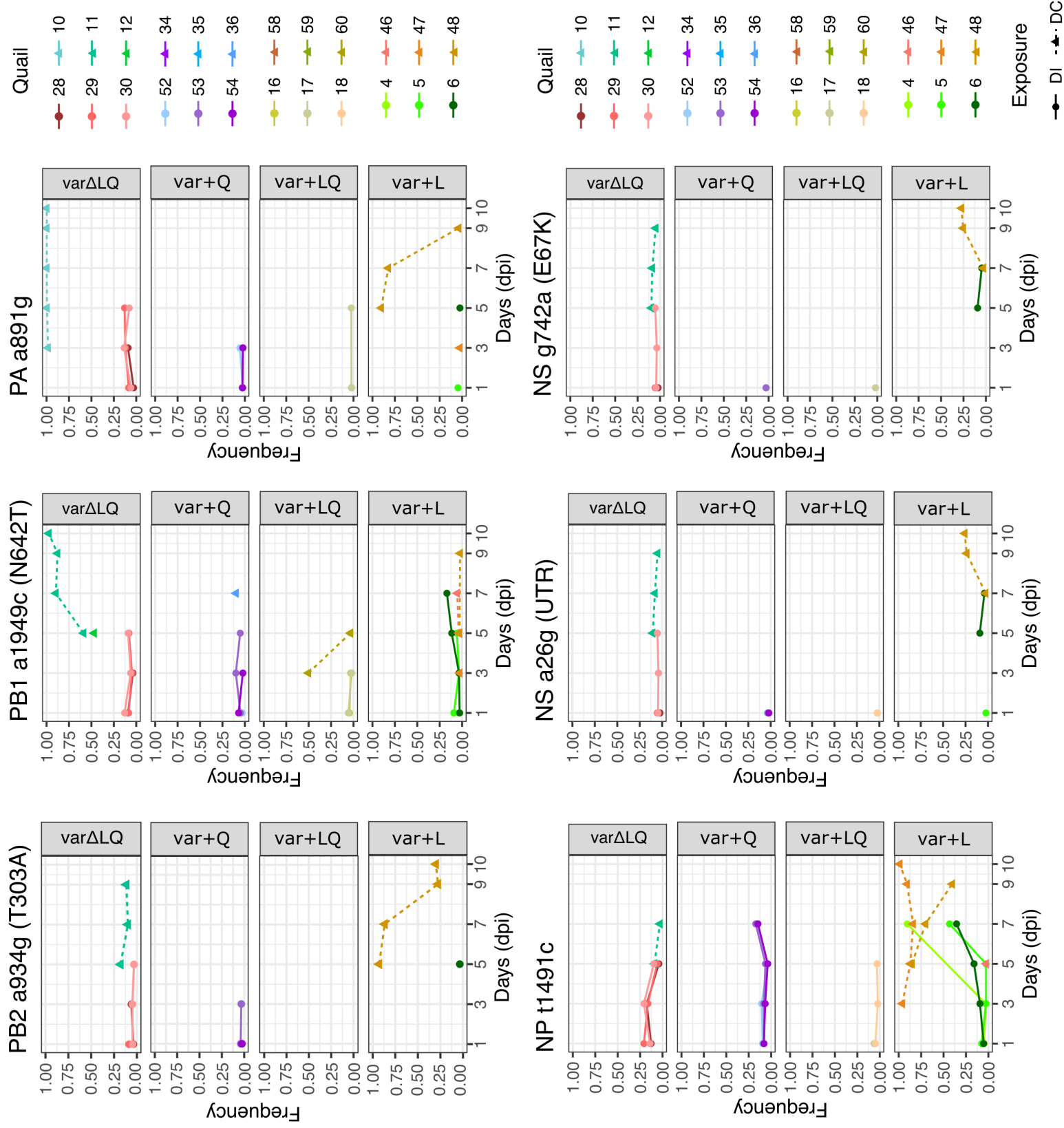

Supplement: veac001_Supp [file veac001_supp.zip › SuppFigure 8 .pdf]

— HA L216    — PA E26L

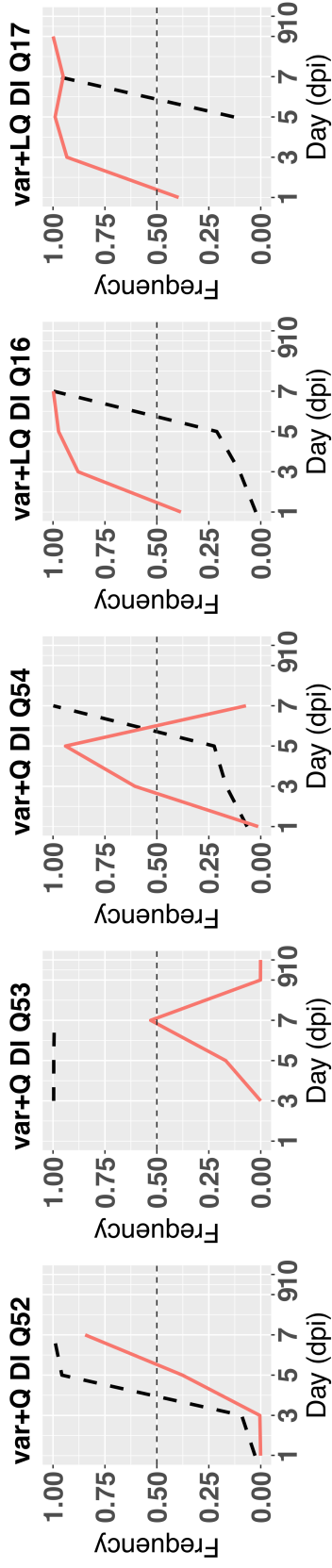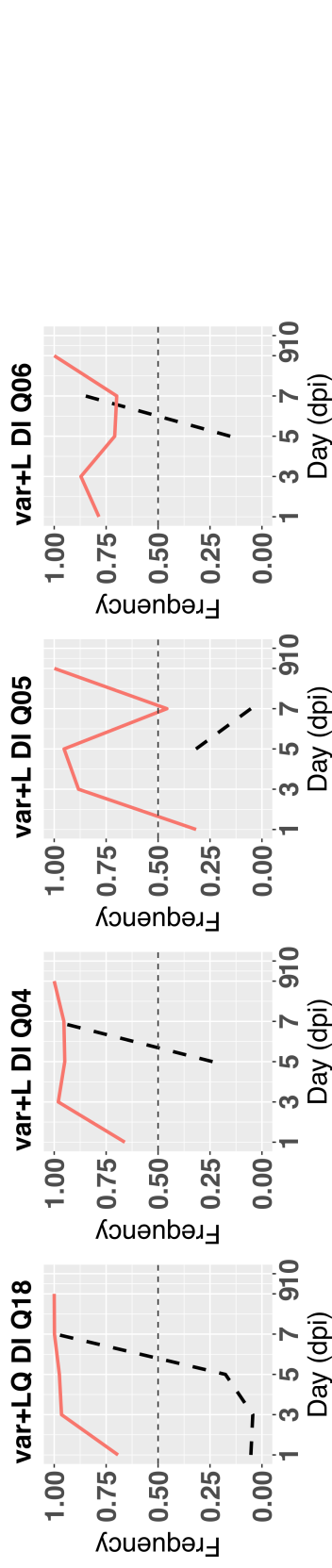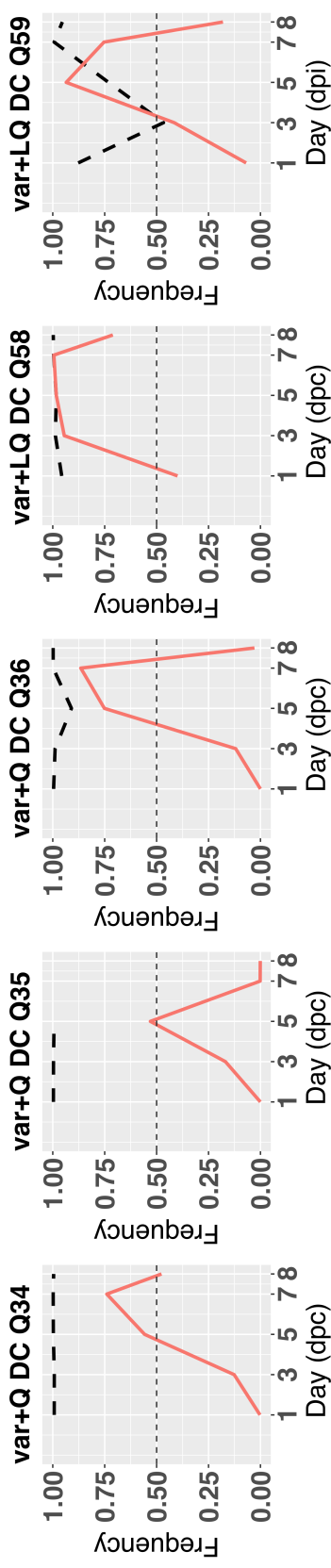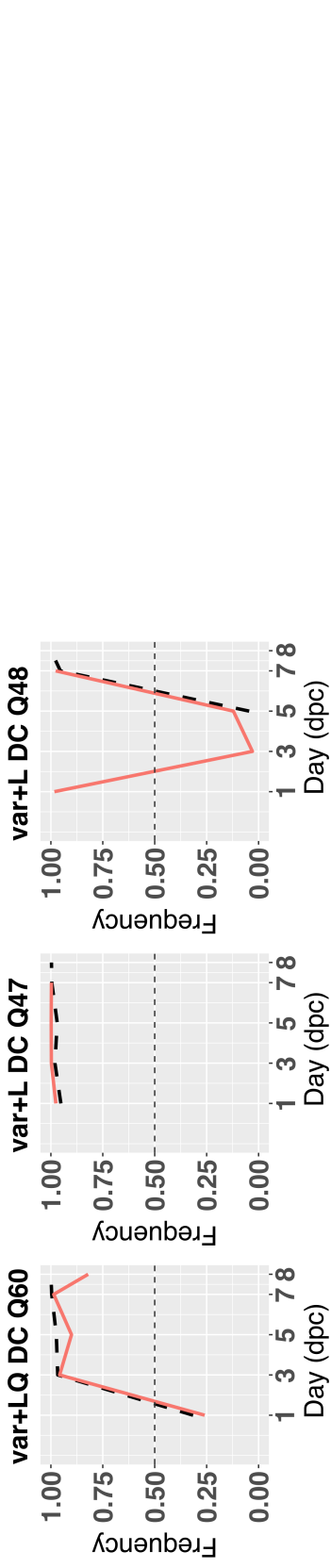

Supplement: veac001_Supp [file veac001_supp.zip › SuppFigure 9.pdf]
